# Improved Formulation of ^224^Ra-Labeled Calcium Carbonate Microparticles by Surface Layer Encapsulation and Addition of EDTMP

**DOI:** 10.3390/pharmaceutics13050634

**Published:** 2021-04-29

**Authors:** Ruth Gong Li, Kim Lindland, Sandra Karen Tonstad, Tina Bjørnlund Bønsdorff, Asta Juzeniene, Sara Westrøm, Roy Hartvig Larsen

**Affiliations:** 1 Oncoinvent AS, 0484 Oslo, Norway; lindland@oncoinvent.com (K.L.); tonstad@oncoinvent.com (S.K.T.); bonsdorff@oncoinvent.com (T.B.B.); westrom@oncoinvent.com (S.W.); sciencons@gmail.com (R.H.L.); 2 Institute of Clinical Medicine, University of Oslo, 0316 Oslo, Norway; 3 Department of Radiation Biology, Institute of Cancer Research, The Norwegian Radium Hospital, Oslo University Hospital, 0379 Oslo, Norway; asta.juzeniene@rr-research.no

**Keywords:** radiopharmaceutical, radionuclide therapy, alpha therapy, alpha emitter, calcium carbonate, microparticles, radium, peritoneal carcinomatosis

## Abstract

Radium-224-labeled CaCO_3_ microparticles have been developed to treat peritoneal carcinomatosis. The microparticles function as carriers of ^224^Ra, facilitating intraperitoneal retention of the alpha-emitting radionuclide. It was necessary to control the size of microparticles in suspension over time and introduce a sterilization process for the clinical use of the radiopharmaceutical. Ethylenediamine tetra(methylene phosphonic acid) (EDTMP) was investigated as a stabilizing additive. The possibility of encapsulating the radiolabeled microparticles with an outer surface layer of CaCO_3_ for the improved retention of radioactivity by the carrier was studied. This work evaluated these steps of optimization and their effect on radiochemical purity, the biodistribution of radionuclides, and therapeutic efficacy. An EDTMP concentration of >1% (*w*/*w*) relative to CaCO_3_ stabilized the particle size for at least one week. Without EDTMP, the median particle size increased from ~5 µm to ~25 µm immediately after sterilization by autoclaving, and the larger microparticles sedimented rapidly in suspension. The percentage of adsorbed ^224^Ra progeny ^212^Pb increased from 56% to 94% at 2.4–2.5% (*w*/*w*) EDTMP when the ^224^Ra-labeled microparticles were layer-encapsulated. The improved formulation also resulted in a suitable biodistribution of radionuclides in mice, as well as a survival benefit for mice with intraperitoneal ovarian or colorectal tumors.

## 1. Introduction

**Figure 1:**
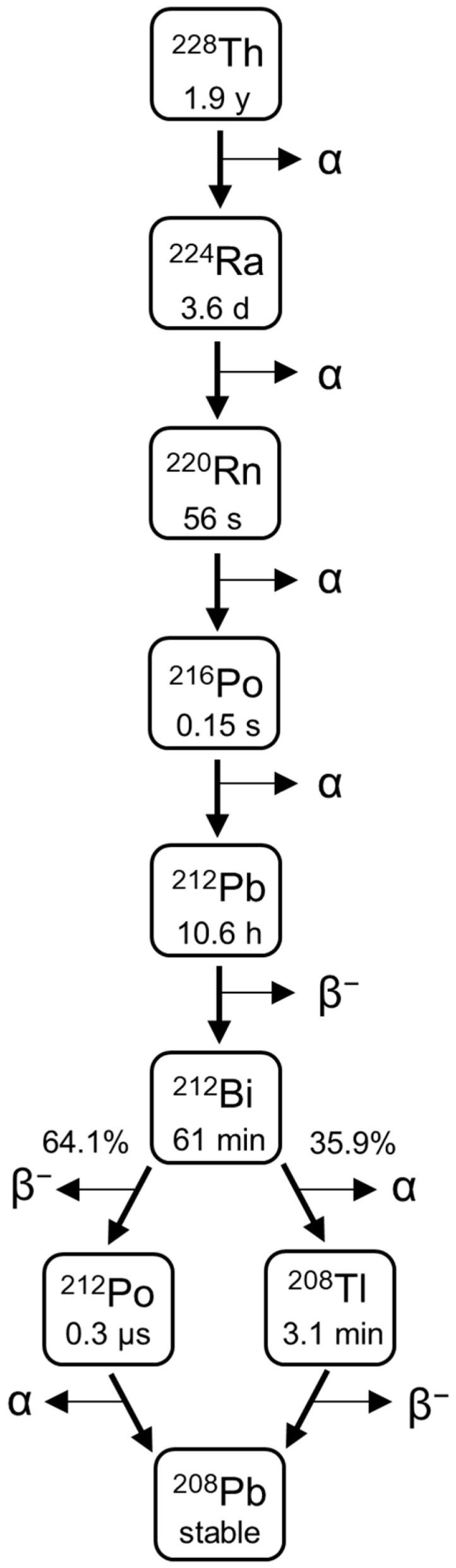
Decay chain of ^224^Ra from parent ^228^Th.

Effective treatment of cancers with peritoneal dissemination by cytoreductive surgery is challenging because of the presence of residual tumor cells and micrometastases. The eradication of small intraperitoneal (i.p.) tumors and single cells can be achieved through the use of highly energetic and short-range alpha radiation in combination with a carrier compound that facilitates i.p. containment of the radioactive payload. Radium-224-labeled calcium carbonate microparticles (^224^Ra-CaCO_3_ MPs) in suspension were developed according to this concept [1], and their therapeutic potential was evaluated in mice with i.p. ovarian cancer [2,3,4]. As a medically promising alpha-emitter, ^224^Ra is a radionuclide with a convenient half-life of 3.6 days. It has more than 90% of its decay energy associated with alpha emissions; ^224^Ra and its progenies emit four alpha particles in total (Figure 1). Further, ^224^Ra can be adsorbed by CaCO_3_ MPs, which are suitable carriers because CaCO_3_ is nontoxic and biodegradable. We previously demonstrated the importance of the CaCO_3_ MPs as a carrier compound by comparing ^224^Ra-CaCO_3_ MPs to free radium-224 dichloride (^224^RaCl_2_) when both were administered i.p. The ^224^Ra-CaCO_3_ MPs resulted in increased i.p. retention of ^224^Ra [1] and extended the survival of mice with tumors [3]. This radiopharmaceutical (Radspherin) is currently being tested in two ongoing clinical phase I trials, as a postoperative treatment to combat the remaining micrometastatic tumors following the complete cytoreductive surgery of peritoneal carcinomatosis originating from ovarian and colorectal cancer [5,6].

One important factor in terms of product stability during the 7-day shelf-life of the radiopharmaceutical is preserving the particle size. The particle size itself influences the ability of MPs to remain suspended, as larger particles settle faster. Calcium carbonate can exist in three different crystalline polymorphs: vaterite, aragonite, and calcite. These different forms have characteristic morphologies: vaterite is typically present as spherical particles, aragonite as needle-like structures, and calcite as rhombohedral particles [7,8]. The thermodynamic stability of the polymorphs also varies; calcite is the only stable form. The dissolution and subsequent reprecipitation of CaCO_3_ in aqueous solution, a process known as recrystallization, causes a transformation from metastable vaterite or aragonite to stable calcite [9]. Moreover, recrystallization causes the growth of individual CaCO_3_ particles. Larger particles grow at the expense of dissolving smaller ones, in a process known as Ostwald ripening [10,11], or the smaller particles may aggregate into larger particles [11]. This leads to increasing particle diameter with time, especially at elevated temperature [11]. Therefore, additives that inhibit recrystallization are necessary to control the size of ^224^Ra-CaCO_3_ MPs, which ensures a stable and dispersed suspension of MPs over time.

Various compounds ranging from small molecules to (bio)polymers have been reported to influence the morphology of CaCO_3_ crystals, e.g., by inhibiting recrystallization [7,10,12,13], and therefore, have a stabilizing effect on crystal structure and/or size. Among these, phosphonic acid derivatives, or phosphonates, are interesting candidates. In relation to CaCO_3_, these compounds have been used in industrial water management to prevent scale formation attributed to CaCO_3_ precipitating on surfaces [14]. It has been proposed that the phosphonates inhibit CaCO_3_ nucleation, adsorb to and block crystal growth sites, distort the crystal lattice, and change the surface charge of crystals [14,15]. The phosphonate ethylenediamine tetra(methylene phosphonic acid) (EDTMP) can retard the transformation from vaterite to calcite [16]. Furthermore, the calcium binding property of phosphonates can be exploited therapeutically due to their consequential skeletal accumulation in diseases such as osteoporosis, where bone resorption by osteoclasts is inhibited by bisphosphonates [17].

Phosphonates have strong chelation properties toward many divalent metal ions, including radiometals [18]. Therefore, in combination with their skeletal targeting, phosphonate-based radiopharmaceuticals have been developed to both diagnose and relieve pain from skeletal metastases [18]. Samarium lexidronam (^153^Sm-EDTMP) is approved worldwide for the pain relief of osteoblastic metastatic bone lesions. While radium itself is inherently a bone-seeker, EDTMP has been used to increase the proportion of daughter nuclides ^212^Pb and ^212^Bi delivered to the bone in mouse models [19,20]. However, if the goal is to ensure radionuclide accumulation at other target sites, the complexation and bone-seeking properties may be problematic. In our application, the CaCO_3_ MPs retain ^224^Ra and progeny, reducing the extraperitoneal release of ^224^Ra and therefore, the level of ^224^Ra in the skeleton [1]. For any stabilizing additive, its influence on the ability of CaCO_3_ MPs to retain radionuclides, i.e., radiochemical purity (RCP), must be clarified.

The aim of this work was to evaluate the crystal growth inhibitor and chelator EDTMP as an additive in suspensions of ^224^Ra-CaCO_3_ MPs in order to determine both its ability to stabilize the particle size during the shelf-life of the product and any potential negative effect on RCP, the in vivo biodistribution of radionuclides, or therapeutic efficacy.

## 2. Materials and Methods

### 2.1. Producing ^224^Ra-CaCO_3_ MPs

Calcium carbonate MPs were produced by spontaneous precipitation according to a previously reported procedure [1,3,21], with the exception of the drying process; the collected precipitate was dried under vacuum at 100 °C for 1 h. Radiolabeling was also performed as described earlier [3], with a few modifications. Radium-224 was extracted from a generator consisting of ^228^Th (Oak Ridge National Laboratory, Oak Ridge, TN, USA) that was either immobilized on a TrisKem Actinide Resin (TrisKem International, Bruze, France) and eluted in 1 M HCl (Suprapur, Merck Group, Darmstadt, Germany) or temporarily immobilized on a Dowex anion exchange resin (Sigma-Aldrich, St. Louis, MO, USA) and then eluted in 0.5 M HNO_3_ (PlasmaPURE Plus, SCP Science, Baie-d’Urfé, QC, Canada) and 80% methanol (Merck Group, Darmstadt, Germany) before evaporation to dryness, which was followed by dissolution in 1 M HCl. In the latter case, the dissolved residue was run through a TrisKem Actinide Resin. In both cases, 5 M NH_4_OAc (Sigma-Aldrich, St. Louis, MO, USA) and 1 M NaOH (VWR International, Radnor, PA, USA) were added to obtain a pH of 7.5–9 in the final ^224^RaCl_2_ solution to be used for radiolabeling. The ^224^RaCl_2_ solution was sterile filtered prior to labeling. The CaCO_3_ MPs were surface-labeled with ^224^Ra by incubation in the ^224^RaCl_2_ solution (0.1–2.7 kBq per mg of CaCO_3_) in the presence of Ba^2+^ (0.004% (*w*/*w*) relative to CaCO_3_) and SO_4_^2−^ (0.6% (*w*/*w*) relative to CaCO_3_) for the coprecipitation of ^224^Ra. The labeled MPs were then washed once with 0.9% NaCl (Fresenius Kabi AG, Bad Homburg, Germany) before any addition of EDTMP (Tokyo Chemical Industry Co., Ltd., Tokyo, Japan) and final suspension in 0.9% NaCl.

A modified layer-encapsulated ^224^Ra-CaCO_3_ MP was prepared by adding an outer CaCO_3_ layer after labeling in an effort to encapsulate its radioactivity. Calcium carbonate microparticles were first surface-labeled as described earlier. After the removal of the incubation solution, equimolar amounts of Na_2_CO_3_ and CaCl_2_ (Merck Group, Darmstadt, Germany) were added to the labeled MPs under vigorous stirring. This led to an additional precipitation process on the surface of the MPs that would increase the total precipitated CaCO_3_ mass by a factor of 2.2–2.4, corresponding to a 30–33% increase in the MP diameter, assuming precipitation exclusively took place on the surface of existing MPs. In some cases, additional Ba^2+^ and SO_4_^2−^ were added during the precipitation process to account for the increase in total CaCO_3_ mass to final relative concentrations of 0.004% (*w*/*w*) and 0.6% (*w*/*w*), respectively. The encapsulated and labeled MPs were washed once with 0.9% NaCl before EDTMP was added, and the MPs were suspended in 0.9% NaCl.

In some cases, where radioactivity was deemed unimportant for the study outcome, CaCO_3_ MPs were either suspended directly in saline (“unlabeled”) or mock-labeled. As for the unlabeled MPs, mock-labeling resulted in a nonradioactive suspension of CaCO_3_ MPs but involved the same preparation steps and reagents as for the radiolabeled and layer-encapsulated MPs, except for the use of 0.9% NaCl in place of a solution of ^224^RaCl_2_.

In all cases, the MPs were suspended in 0.9% NaCl, sealed in a crimp neck glass headspace vial, and sterilized in an autoclave at 121 °C for 20 min. The suspension cooled to room temperature before further handling.

The remaining sections will distinguish surface-labeled MPs from layer-encapsulated MPs for clarity, despite the fact that both were surface-labeled initially.

### 2.2. Particle Size Measurements

The size of unlabeled, mock-labeled, and radiolabeled CaCO_3_ MPs in suspension with varying concentrations of EDTMP was measured with laser diffraction (Mastersizer 3000, Malvern Instruments Ltd., Worcestershire, UK). The unautoclaved CaCO_3_ MPs used as raw material for radio- and mock-labeling were used as a reference by dispersing a small amount of dried CaCO_3_ MPs in water and ultrasonicating to disperse. Size stability over time was evaluated in radiolabeled CaCO_3_ MPs by measuring after seven days of storage at room temperature; surface-labeled MPs were compared with layer-encapsulated MPs.

### 2.3. Influence of EDTMP on the Sedimentation Rate of MPs

The ability of MPs to remain suspended in solution was evaluated by the sedimentation rate, which was investigated through the visual inspection of samples and by evaluating the turbidity of different suspensions of nonradioactive mock-labeled CaCO_3_ MPs, with and without EDTMP. Turbidity was assessed by diluting the CaCO_3_ MP suspension with water (water for injection) and then measuring the change in optical density at a wavelength of 800 nm over 30 min using a spectrophotometer (Hitachi U-1900, Hitachi High-Tech, Tokyo, Japan). The 800 nm wavelength was chosen to reduce potential light absorbance by CaCO_3_ and improve light scattering by particles. A decrease in optical density with time is, therefore, directly related to decreased light scattering by MPs and thereby, decreased turbidity of the sample due to sedimentation.

### 2.4. Influence of EDTMP on Radiochemical Properties

The intrinsic product stability of ^224^Ra-CaCO_3_ MPs during shelf-life was assessed by measuring the radiochemical purity and comparing surface-labeled MPs to layer-encapsulated MPs. Radiochemical purity was defined as the percentage of radionuclides retained on the MPs after a certain period. A small aliquot of suspension was separated into MP fraction P and supernatant fraction S by centrifugation. The percentage radiochemical purity, % RCP, was defined as the proportion of radioactivity in the P fraction: CPM(P)/CPM(P+S), with CPM denoting counts per minute. The radioactivity of the two fractions was measured separately using a Hidex Automatic Gamma Counter (Hidex Oy, Turku, Finland). Sample tubes with air-tight lids were used to avoid potential ^220^Rn escape from the samples [4]. Radioactivity of ^212^Pb was quantified by counts in the 60–110 keV window [22]. For ^224^Ra, radioactivity was determined indirectly by assuming a transient equilibrium between ^224^Ra and progeny ^212^Pb after allowing the two fractions to decay for at least two days and then measuring ^212^Pb activity in the 65–345 keV window, in which gamma energy and X-rays mainly originated from this daughter. Sampling and measurement were repeated after up to seven days of storage at room temperature to evaluate the stability of ^212^Pb and ^224^Ra % RCP over time.

The complexation between the released ^212^Pb from MPs and EDTMP in the solution was evaluated in the liquid phase of different variants of ^224^Ra-CaCO_3_ MPs. The liquid fraction was first separated from the MPs by centrifugation. The degree of ^212^Pb-EDTMP complexation in the obtained supernatant was then measured using instant thin-layer chromatography (ITLC) strips (Tec-Control Chromatography Systems #150-772, Biodex Medical Systems, Inc., Shirley, NY, USA). Chelated ^212^Pb will migrate with the mobile phase in this system, while most (>90%) unbound ^212^Pb^2+^ will remain at the origin line, allowing for the evaluation of ^212^Pb-EDTMP complexation. Water (pharmaceutical grade) or 0.9% NaCl was used as the mobile phase, and the strips were cut in half after the solvent front had reached the top line. The radioactivity of ^212^Pb in the two parts was measured with a Hidex Automatic Gamma Counter as described earlier. The degree of chelation was defined by the proportion of migrated ^212^Pb in the liquid fraction of ^224^Ra-CaCO_3_ MPs and was quantified by subtracting the unspecific migration of free ^212^Pb^2+^ in a 0.9% NaCl solution without EDTMP. Equation (1) describes the percentage chelation; A_212Pb-EDTMP_ denotes the measured activity in the supernatant of ^224^Ra-CaCO_3_ MPs; A_212Pb_ denotes the measured activity of free ^212^Pb^2+^ in the 0.9% NaCl solution, and *m* and *o* denote the two parts of the ITLC strip; *m* denotes migration with the mobile phase and *o* denotes the origin line.

(1) $$\%\;\mathrm{chelation}=\left(\frac{A_{212\mathrm{Pb}-\mathrm{EDTMP},\;m}\;}{A_{212\mathrm{Pb}-\mathrm{EDTMP},\;m}{\;+\;A}_{212\mathrm{Pb}-\mathrm{EDTMP},\;o}}\;-\;\frac{A_{212\mathrm{Pb},\;m}}{A_{212\mathrm{Pb},\;m}{\;+\;A}_{212\mathrm{Pb},\;o}}\right)\times 100\%$$

### 2.5. Biodistribution

The biodistribution of layer-encapsulated ^224^Ra-CaCO_3_ MPs with added EDTMP was evaluated in institutionally bred nontumor-bearing female athymic nude mice (Hsd: Athymic Nude-*Foxn1^nu^*, Department of Comparative Medicine, The Norwegian Radium Hospital, Oslo University Hospital, Oslo, Norway). Calcium carbonate microparticles were labeled and autoclaved as described earlier. The impact of mass dose (mg dose) was considered by testing doses ranging from 1–12 mg CaCO_3_ and 6–18 kBq by creating dilutions with an isotonic infusion solution (Plasmalyte, Baxter International Inc., Deerfield, IL, USA). One day after a single i.p. administration, the mice were euthanized by cervical dislocation, and tissue samples were obtained to measure radioactivity. Three standard samples corresponding to 25–50% of the administered dose of each treatment were used to determine the injected radioactivity dose. The radioactivity of ^212^Pb and ^224^Ra of tissue and standard samples was measured using a gamma counter as described above, from which the percentage injected dose per gram tissue (% ID/g) was calculated. Correction for decay and/or ingrowth of ^224^Ra and ^212^Pb was not performed in the calculation of the % ID/g for two reasons: firstly, standard samples and tissue samples were counted with less than 2–3 h time interval (i.e., 3% of the half-life of ^224^Ra), and secondly, error propagation as a result of uncertainty in the measurement of ^224^Ra, when the measured activity was close to or below the limit of quantification of the instrument, could be avoided. As a reference for the skeletal accumulation of free ^224^Ra^2+^ one day after i.p. injection, one group of mice received ~30 kBq ^224^RaCl_2_ prepared as described previously [3]. An overview of the experimental groups is provided in Supplementary Table S1.

### 2.6. Therapeutic Efficacy

The therapeutic effect of radiolabeled MPs of different sizes with ^224^Ra adsorbed either on the surface or beneath an outer protective layer was evaluated. Layer-encapsulated ^224^Ra-CaCO_3_ MPs suspended in EDTMP (1% (*w*/*w*) relative to CaCO_3_) and 0.9% NaCl solution were compared to that of the surface-labeled variant suspended in 0.9% NaCl only, by evaluating the survival rate of mice with tumors. To establish i.p. ovarian cancer xenografts, nude mice aged 4–5 weeks were inoculated with 1 million ES-2 cells (ATCC, Wesel, Germany) [2,3,4]. For a syngeneic colorectal cancer model, BALB/c mice (BALB/cAnNRj, Janvier Labs, Le Genest-Saint-Isle, France) aged six weeks were inoculated i.p. with 50,000 CT26.WT cells (LGC Standards, ATCC, Wesel, Germany) in a study managed by Minerva Imaging (Ølstykke, Denmark). In both cases, mice were randomized and received a single i.p. injection of autoclaved ^224^Ra-CaCO_3_ MPs (14–26 kBq, 4–14 mg), or 0.9% NaCl as vehicle control, one day after tumor inoculation. The dosing was selected based on previously tested efficacious doses with former generations of unautoclaved ^224^Ra-CaCO_3_ MPs in the ES-2 model [2,3,4]. An overview of the experimental groups can be found in Supplementary Table S2. Animals were supplied with food and water ad libitum and euthanized by cervical dislocation when reaching predetermined study endpoints, which included rapid body weight loss or severe build-up of ascites. Animals were censored if they lived beyond the timepoint corresponding to three times the median survival time of the longest surviving group.

### 2.7. Statistical Analysis

A statistical analysis of the differences between the experimental groups in the animal studies was performed with GraphPad Prism (Version 8.1.2, GraphPad Software, San Diego, CA, USA). A *t*-test was performed on each pair of experimental groups in the biodistribution study to detect differences in the % ID/g while adjusting the obtained *p*-value to account for multiple comparisons using the Holm–Sidak method. In the studies of therapeutic efficacy, differences in the survival curves were analyzed using the Gehan–Breslow–Wilcoxon method while adjusting the obtained *p*-values using the Holm–Sidak method. In both cases, an adjusted *p* < 0.05 was considered statistically significant.

## 3. Results

### 3.1. Stabilization of Microparticle Size; Influence of EDTMP and Layer Encapsulation

**Figure 2:**
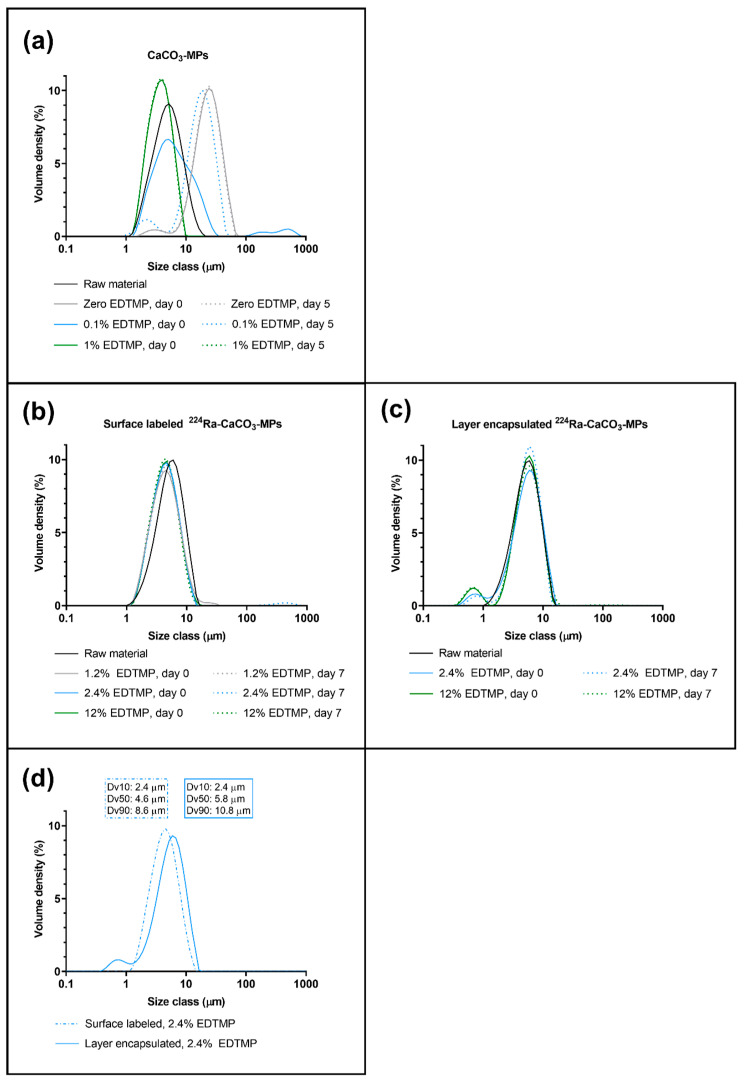
Size distribution of CaCO_3_ microparticles. (**a**) Unlabeled, nonradioactive CaCO_3_ MPs suspended in saline and autoclaved on day zero with varying EDTMP % (*w*/*w*) compared with the unautoclaved CaCO_3_ MPs used as raw material. (**b**) Surface ^224^Ra-labeled CaCO_3_ MPs with varying EDTMP % (*w*/*w*) compared with the unautoclaved CaCO_3_ MPs used as raw material. (**c**) Autoclaved and layer-encapsulated ^224^Ra surface-labeled CaCO_3_ MPs with varying EDTMP % (*w*/*w*) compared with the unautoclaved CaCO_3_ MPs used as raw material. (**d**) Comparison of surface-labeled and layer-encapsulated surface-labeled MPs, excerpts from (**b**,**c**) Dv: volumetric diameter, with Dv10 being the 10th percentile, etc.

As an additive, EDTMP was studied with the purpose of obtaining size control over ^224^Ra-CaCO_3_ MPs. When no EDTMP was present, the median (volume-based) diameter of MPs increased from 5 µm in the raw material to 25 µm immediately after autoclaving (day zero) of the suspension but remained stable for at least five days thereafter (Figure 2a, Table 1). A concentration of 0.1% (*w*/*w*) EDTMP relative to CaCO_3_ was able to retain the size of MPs on day zero, however, five days later, the median diameter had grown to 18 µm. A concentration of 1% (*w*/*w*) EDTMP relative to CaCO_3_ was necessary to stabilize the MP size for at least five days. This concentration resulted in a slight shift of the size distribution to a smaller size as compared with the CaCO_3_ MPs used as raw material; this is because of the improved dispersion of the suspension.

The EDTMP concentration of 1% (*w*/*w*) relative to CaCO_3_ retained the size of autoclaved surface ^224^Ra-labeled CaCO_3_ MPs for at least seven days, with no change in particle diameter when the concentration was increased to 12% (*w*/*w*) (Figure 2b, Table 1).

For the autoclaved layer-encapsulated MPs that were either surface-labeled with ^224^Ra or mock-labeled, a minimum EDTMP concentration of 2.4% (*w*/*w*) relative to CaCO_3_ was necessary to disperse MPs in the autoclaved suspension in these experiments. The size remained stable for at least seven days, and increasing the EDTMP concentration to 12% (*w*/*w*) did not influence MP size (Figure 2c, Table 1). A minor population of submicrometer particles was detected, suggesting that the process of layer encapsulation causes the formation of a small volume of new particles in addition to creating layers on pre-existing particles. The direct comparison of the surface-labeled MPs with the encapsulated ones showed that both the 50th and 90th percentile of MP-size increased by ~20% on average (*n* = 4) at 2.4–2.5% (*w*/*w*) EDTMP, which supports the formation of an outer layer of CaCO_3_ on the radiolabeled MPs (Figure 2d, Table 1).

**Figure 3:**
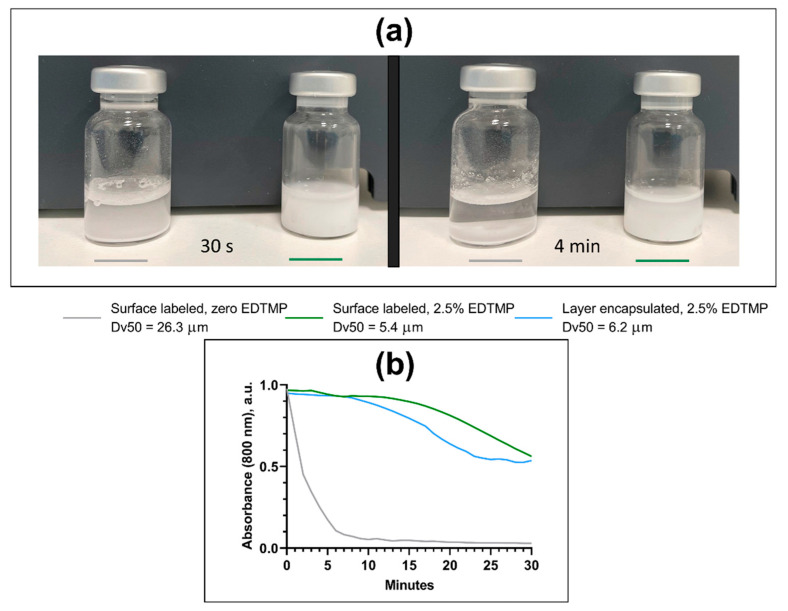
Sedimentation rate of nonradioactive autoclaved CaCO_3_ microparticles of different sizes. (**a**) Comparison of mock surface-labeled MPs suspended in 0.9% NaCl with and without EDTMP, after the suspension was allowed to sit for 30 s and 4 min, respectively. (**b**) Assessment of the turbidity of suspended CaCO_3_ MPs of different sizes, all mock surface-labeled, and layer-encapsulated MPs are indicated by the blue line. Dv: volumetric diameter, with Dv50 being the 50th percentile.

When EDTMP was present at 2.4–2.5% (*w*/*w*), the smaller CaCO_3_ MPs (median diameter of 5–6 µm) remained suspended for a considerably longer time compared to CaCO_3_ MPs without EDTMP (median diameter of 25–26 µm); this was visible with the naked eye immediately after the suspensions were autoclaved (Figure 3a). The large difference in the sedimentation rate between samples with and without EDTMP was also detected in a turbidity assessment (Figure 3b). Little variation was observed for MP suspensions with EDTMP, but the measurement indicated a slightly increased sedimentation rate of the layer-encapsulated MPs compared to the surface-labeled MPs between 10 and 30 min (Figure 3b).

A light microscope showed that EDTMP preserved the spherical morphology of CaCO_3_ MPs after autoclaving, as the MPs would otherwise recrystallize into cuboid structures (Supplementary Figure S1).

### 3.2. Radiochemical Properties Depending on EDTMP and Layer Encapsulation

**Figure 4:**
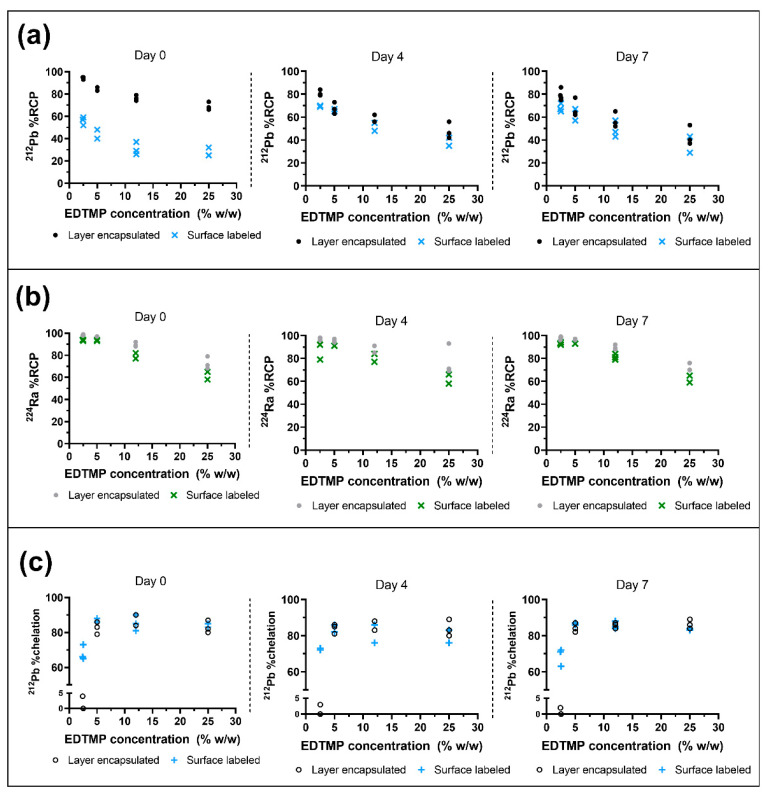
Radiochemical analysis of adsorbed ^212^Pb and ^224^Ra on CaCO_3_ microparticles (MPs) with varying EDTMP concentrations. Symbols represent independent samples and EDTMP concentration is relative to CaCO_3_. (**a**) Percentage of adsorbed ^212^Pb (% RCP) on the MPs on different days after labeling, surface-labeled MPs vs layer-encapsulated surface-labeled MPs. (**b**) Percentage of adsorbed ^224^Ra on the MPs (% RCP) on different days after labeling, surface-labeled MPs vs layer-encapsulated surface-labeled MPs. (**c**) Percentage of ^212^Pb-EDTMP in the liquid phase after subtracting unspecific migration by ^212^Pb^2+^ in 0.9% NaCl in the ITLC setup on different days after labeling, surface-labeled MPs vs layer-encapsulated surface-labeled MPs.

The decay of ^224^Ra results in ^212^Pb, which is known to form a complex with EDTMP [19,20]. For this reason, it was suspected that the presence of EDTMP could influence the fraction of ^212^Pb adsorbed on CaCO_3_ MPs (% RCP) versus released ^212^Pb in the liquid phase. For a given EDTMP concentration in the suspension of ^224^Ra-CaCO_3_ MPs, % RCP on the same day as labeling (day zero) was highest for the layer-encapsulated MP variant when compared to surface-labeled MPs in all cases. For both variants, there was a trend of decreasing RCP with increasing EDTMP concentration (Figure 4a). Increased ^212^Pb % RCP was observed for surface-labeled MPs from day zero to day four for all of the tested EDTMP concentrations, indicating the readsorption of ^212^Pb from decayed ^224^Ra. However, the % RCP remained relatively stable for the layer-encapsulated MPs, though with the highest value on day zero (Figure 4a). The % RCP of ^224^Ra appeared unaffected by the outer CaCO_3_ layer on MPs, but a higher fraction of radionuclides remained on both MP variants for the two lowest EDTMP concentrations (Figure 4b). The RCP remained stable for at least seven days; there was no detectable difference between the two MP variants for a given EDTMP concentration for ^224^Ra (Figure 4b).

Radioactivity measurements of ITLC strips were performed to evaluate the degree of complexation between unbound ^212^Pb^2+^ and EDTMP in the liquid phase of ^224^Ra-CaCO_3_ MPs, correcting for unspecific migration by ^212^Pb^2+^ in 0.9% NaCl. The analysis revealed that the fraction of chelated ^212^Pb increased for EDTMP concentrations ≥5% (*w*/*w*) as compared to 2.4–2.5% (*w*/*w*) and remained stable for at least one week (Figure 4c). The significance of the layer encapsulation was only visible at the lowest EDTMP concentration of 2.4–2.5% (*w*/*w*) with 0–4% chelation, compared to 63–73% chelation in the surface-labeled variant.

### 3.3. Biodistribution of Layer-Encapsulated ^224^Ra-CaCO_3_ MPs with EDTMP

**Figure 5:**
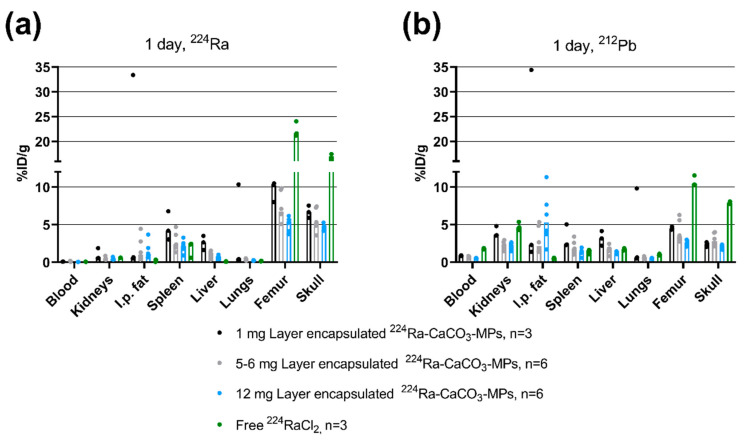
Biodistribution of layer-encapsulated ^224^Ra surface-labeled CaCO_3_ microparticles one day after i.p. injection. Bars represent the median and symbols represent individual animals. Animals that were treated with equal or near-equal mass doses have been pooled into one group. (**a**) Percentage injected dose per gram tissue of ^224^Ra. (**b**) Percentage injected dose per gram tissue of ^212^Pb from the same samples as in (**a**). There is missing data for ^224^Ra and ^212^Pb for one skull in the ^224^RaCl_2_ group and for ^212^Pb for one blood sample in the 12 mg group due to instrument errors.

The biodistribution of 1–12 mg layer-encapsulated ^224^Ra-CaCO_3_ MPs with 1.2–2.5% (*w*/*w*) EDTMP relative to CaCO_3_ was evaluated one day after i.p. administration in mice. A significant decrease in % ID/g of both ^224^Ra and ^212^Pb in the femur and skull was observed for all of the tested doses (*p* < 0.0050) as compared to i.p. administration of free ^224^Ra^2+^ (Figure 5a,b, Supplementary Table S3). Varying the mass dose had little effect on biodistribution, apart from a decrease in skeletal % ID/g of ^224^Ra when injecting 12 mg as compared to 1 mg (*p* < 0.0075, Supplementary Table S3). Low or modest levels of ^212^Pb were detected in the skeleton at all tested ^224^Ra-CaCO_3_ MP doses (Figure 5b). Variability in the % ID/g in the i.p. fat was attributed to a technical difficulty when removing small MP residues from these tissue samples.

### 3.4. Influence on Therapeutic Efficacy by Particle Size, Layer Encapsulation, and EDTMP

**Figure 6:**
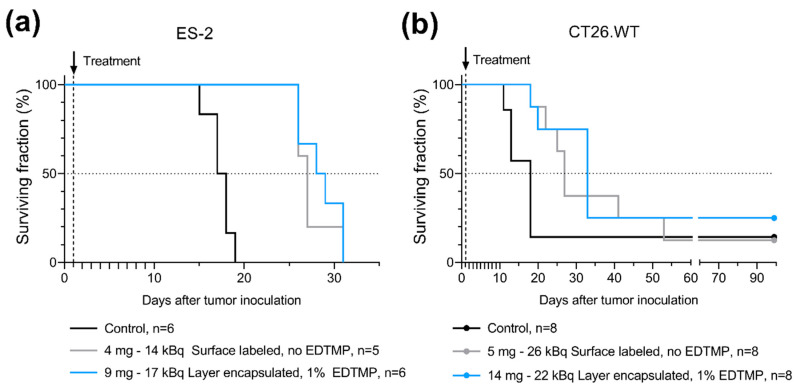
Therapeutic effect of ^224^Ra-labeled microparticles; surface-labeled microparticles vs layer-encapsulated surface-labeled microparticles. (**a**) Kaplan–Meier curves of nude mice inoculated with ES-2 cells on day zero. (**b**) Kaplan–Meier curves of BALB/c mice inoculated with CT26.WT cells on day zero. There are censored mice on day 99, corresponding to three times the median survival time of the longest surviving group at which the study was terminated; one mouse is censored on the black and grey curves while two mice are censored on the blue curve.

The effect of the MP size and layer-encapsulation of radiolabeled MPs on therapeutic efficacy was assessed in mice with i.p. xenograft ovarian cancer or i.p. syngeneic colorectal cancer. Layer-encapsulated ^224^Ra-CaCO_3_ MPs with 1% (*w*/*w*) EDTMP relative to CaCO_3_ and a median diameter of 7–9 µm were compared with a surface-labeled variant without EDTMP added and a median diameter of 9–23 µm. The latter resembled the ^224^Ra-CaCO_3_ MPs reported previously [3], apart from the terminal sterilization by autoclaving, which was not performed in previous work. A survival benefit was observed in both tumor models when they were treated with the two formulations as compared to the control group (Figure 6, Supplementary Table S4, highest *p*-values: *p* = 0.0068 for the ES-2 model and *p* = 0.044 for CT26.WT). The two variants performed almost equally in terms of median survival time in both tumor models (27 and 29 days for ES-2, 27 and 33 days for CT26.WT). Hence, no statistical difference was observed between the two ^224^Ra-CaCO_3_ MP treatments (*p* = 0.4477 for ES-2, *p* = 0.6331 for CT26.WT). All animals were euthanized exclusively at disease-related endpoints, including ascites development and/or palpable tumors.

## 4. Discussion

This work has shown that the size of ^224^Ra-CaCO_3_ MPs in suspension can be stabilized for at least one week by adding the recrystallization inhibitor EDTMP to the suspension and that the percentage of the daughter nuclide ^212^Pb retained on the MPs can be increased by an outer encapsulating CaCO_3_ surface layer.

Additives that inhibit recrystallization are necessary to prevent the growth of CaCO_3_ MPs in suspension, while the particle size itself is important for the MP to remain suspended. The present work considered only autoclaved suspensions of MPs, contrasting with our previously published work on the ^224^Ra-CaCO_3_ MPs [1,2,3,4], because a sterilization procedure is compulsory for a radiopharmaceutical intended for clinical use. The growth of CaCO_3_ MPs with no EDTMP added was detected immediately after the suspension was autoclaved. Even though the increased particle diameter remained stable thereafter, the particles sedimented fast and the suspension was difficult to disperse. The size distribution of CaCO_3_ MPs remained constant from unlabeled raw material to radiolabeled MPs in suspension when EDTMP was added. It is important to note that the sedimentation rate was substantially reduced when the particle size was decreased and that the smaller MPs were easier to disperse and handle, resulting in a significant advantage in terms of clinical administration of the product.

After obtaining size control via EDTMP, layer encapsulation was introduced to optimize the radiochemical properties of the ^224^Ra-CaCO_3_ MPs, as EDTMP is known to chelate ^212^Pb and other divalent metals [18,19,20]. The presence of an outer CaCO_3_ layer on CaCO_3_ MPs that had already been surface-labeled was supported by comparing their size distribution to that of CaCO_3_ MPs that were only surface-labeled. The slight increase in MP diameters from the surface-labeled analog did not influence their ability to disperse. However, the additional precipitation process resulted in the formation of a small volume of submicrometer CaCO_3_ particles that could only be detected after layer-encapsulation.

It was suspected that complexation of ^212^Pb with EDTMP would result in a decrease in MP-bound ^212^Pb, which could potentially lead to the undesired release of ^212^Pb to systemic circulation in vivo and localization of ^212^Pb-EDTMP to the skeleton. The largest difference between surface-labeled and layer-encapsulated ^224^Ra-CaCO_3_ MPs with EDTMP was detected in their ability to retain ^212^Pb. At 2.4–2.5% (*w*/*w*) EDTMP relative to CaCO_3_, the ^212^Pb % RCP of layer-encapsulated ^224^Ra-CaCO_3_ MPs was highest on day zero (94%) and remained above 79% on average over the course of seven days. The cumulative amount of the chemically equivalent stable daughter nuclide ^208^Pb (Figure 1) adsorbed on the MPs increases with time (Supplementary Figure S2), although this seems to have little effect on the adsorption of ^212^Pb. For the surface-labeled variant at this EDTMP concentration, % RCP of ^212^Pb was only 56% on day zero, with an increase to 70% on day four. The increased adsorption of ^212^Pb from day zero to day four was a general observation for surface-labeled MPs. For both MP variants, the persistence of adsorbed ^212^Pb over time is a result of either retention of ^224^Ra daughters after decay, reassociation of released ^212^Pb, or a combination of the two. We have previously shown that a certain emanation of the gaseous ^220^Rn, the parent of ^212^Pb, occurs from ^224^Ra-CaCO_3_ MPs, with ^212^Pb being substantially readsorbed by the MPs [4]. For the optimized formulation herein, readsorption may also be mediated by EDTMP, as released ^212^Pb^2+^ is sequestered by EDTMP at sufficient EDTMP concentration. The known complexation property of EDTMP with both ^212^Pb and calcium indicates that it is also possible for the ^212^Pb-EDTMP complex to associate with the MPs. To test this hypothesis, adsorption of ^212^Pb on nonradioactive mock-labeled CaCO_3_ MPs, both with and without layer-encapsulation, was evaluated after the addition of a solution of ^212^Pb-EDTMP. It was found that 17–20% of the ^212^Pb-EDTMP adsorbed on the MPs; the adsorption increased to 96% when unbound ^212^Pb^2+^ (^212^PbCl_2_) was added instead (Supplementary Figure S3). The reduced adsorption of ^212^Pb-EDTMP is in line with the general observation that the % RCP of ^212^Pb of both surface-labeled MPs and layer-encapsulated MPs decreased at higher EDTMP concentrations in the MP suspension. The exact distribution of EDTMP in solution versus on the MPs themselves is not known, although it can be argued that the majority is adsorbed on the MPs due to the low presence of chelated ^212^Pb in solution for the layer encapsulated ^224^Ra-CaCO_3_ MPs with 2.4–2.5% (*w*/*w*) EDTMP (Figure 4c).

The stable particle size in combination with the promising radioactivity-retention properties of the layer-encapsulated ^224^Ra-CaCO_3_ MPs with low EDTMP concentration warranted further investigation in animal models. A suitable biodistribution pattern of both ^212^Pb and ^224^Ra was achieved after i.p. administration at 1.2–2.5% (*w*/*w*) EDTMP. The substantially decreased % ID/g of both ^212^Pb and ^224^Ra detected in the skeleton when compared with i.p. injection of ^224^RaCl translates to low levels of radionuclides leaking from the peritoneal cavity. The slightly higher skeletal activity for the 1 mg dose as compared to 5–12 mg is attributed to the difference in specific activity, i.e., activity per CaCO_3_ mass (1–1.8 kBq/mg vs. ~6 kBq/mg), which is in line with previous work [1,3]. Low levels of ^212^Pb were detected in the skeleton as compared to the previously published biodistribution results of ^212^Pb-EDTMP after i.v. administration to mice [19], indicating a limited release of any potential ^212^Pb-EDTMP to systemic circulation. The localization of EDTMP to bone is attributed to its affinity to hydroxyapatite and is dependent on calcium concentration rather than the number of osteoblasts [23]. Hence, the presence of Ca^2+^ from CaCO_3_ may possibly contribute to retaining EDTMP on MPs within the peritoneal cavity, at least within the relatively short half-lives of ^224^Ra and its daughters.

The loading of radionuclides into carrier MPs for tumor radiotherapy is an approach that has historically been used for beta emitters. [24]. For alpha emitters, the incorporation of the nuclide into the bulk of the carrier can be advantageous as a means of retaining recoiling daughter nuclides to prevent unintentional escape and redistribution in vivo. Incorporation of alpha emitters such as ^225^Ra/^225^Ac, and/or ^223^Ra into nanoparticles has been reported in liposomes [25,26], LaPO_4_ [27], LaVO_4_ [28] and hydroxyapatite [29], among others. Further, a study of ^225^Ac-labeled CaCO_3_ MPs was recently published, with the ^225^Ac incorporated into MPs and submicron particles that were coated with a protein and polyphenol on the particle surface as stabilizing agents [30]. Ensuring that the encapsulation does not compromise the already short range of the alpha particles (50–100 µm in tissue) is a prerequisite for alpha emitters to be carried inside MPs. The MP size and the thickness of the encapsulating layer of ^224^Ra-CaCO_3_ MPs are both very small compared to the range of alpha particles and are therefore not expected to significantly limit the range. However, size-dependent sedimentation differences may potentially affect the distribution of the infused microparticles. In this work, the data from studies of mice with tumors was inconclusive in terms of the potential influence of particle size and EDTMP on survival. A survival benefit in mice was observed in two different tumor models after a single i.p. injection, but no statistical difference was detected when the smaller layer-encapsulated ^224^Ra-CaCO_3_ MPs with EDTMP were compared to the larger surface-labeled ^224^Ra-CaCO_3_ MPs without EDTMP.

Ongoing clinical studies (NCT03732768 [5], NCT03732781 [6]) with ^224^Ra-CaCO_3_ MPs (Radspherin) have been initiated on the basis of the promising preclinical data on layer-encapsulated ^224^Ra-CaCO_3_ MPs with 1.2–2.5% (*w*/*w*) EDTMP added.

## 5. Conclusions

To summarize, we have optimized ^224^Ra-CaCO_3_ MP formulation to achieve a suitable product for clinical usage. The addition of EDTMP stabilizes size over time, thereby increasing the ability of MPs to remain suspended, which is important for ease of handling during administration of the product to patients. The layer-encapsulation of the radiolabeled MPs and the addition of 2.4–2.5% (*w*/*w*) EDTMP relative to CaCO_3_ provide suitable radiochemical and biodistribution properties of the radionuclides. The optimized ^224^Ra-CaCO_3_ MPs had a therapeutic effect in the tumor models presented here; antitumor efficacy was not affected by the modification.

## 6. Patents

The presented technology is covered by “Radiotherapeutic particles and suspensions”, patent number US9539346 B1. The privately held company Oncoinvent AS holds intellectual property rights.

## Figures and Tables

**Table 1:** Size distribution of various CaCO_3_ MPs from Figure 2, shown as the volumetric 10th percentile (Dv10), 50th percentile (Dv50), and 90th percentile (Dv90). Day 0 refers to the day of preparing the sample including autoclaving of all samples with the exception of the raw material. na: not applicable.

Compound	EDTMP Concentration (*w*/*w*)%	Particle Size Distribution (Dv10/Dv50/Dv90) on Day 0 (µm)	Particle Size Distribution (Dv10/Dv50/Dv90) on Day 5 or 7 (µm)
CaCO_3_ MP raw material ^(1)^	0	2.5/5.0/9.6	na
CaCO_3_ MPs ^(1)^	0	12.2/24.7/44.5	12/24.2/43.3
CaCO_3_ MPs ^(1)^	0.1%	2.7/6.5/19.6	7.5/18.3/32.8
CaCO_3_ MPs	1%	2.0/3.9/6.8	2.2/3.9/6.7
CaCO_3_ MP raw material ^(2)^	0	2.8/5.7/10.3	na
Surface-labeled ^224^Ra-CaCO_3_ MPs ^(2)^	1.2%	2.4/4.7/9.3	2.4/4.6/8.8
Surface-labeled ^224^Ra-CaCO_3_ MPs ^(2,4)^	2.4%	2.4/4.6/8.6	2.4/4.7/8.9
Surface-labeled ^224^Ra-CaCO_3_ MPs ^(2)^	12%	2.4/4.6/8.6	2.4/4.5/8.3
CaCO_3_ MP raw material ^(3)^	0	2.8/5.7/10.3	na
Layer-encapsulated ^224^Ra-CaCO_3_ MPs ^(3)^	2.4%	2.4/5.8/10.8	2.9/5.9/9.9
Layer-encapsulated ^224^Ra-CaCO_3_ MPs ^(3,4)^	12%	2.6/5.7/10.2	2.5/5.7/10.7

^(1)^ Size distribution shown in Figure 2a, ^(2)^ size distribution shown in Figure 2b, ^(3)^ size distribution shown in Figure 2c, ^(4)^ size distribution shown in Figure 2d.

## Data Availability

The reported data is available from Oncoinvent AS, although there are restrictions on its availability under the license of the presented work, and it is not publicly available. However, data is available from the authors upon reasonable request and with the permission of Oncoinvent AS.

## Supplementary Materials

The following are available online at https://www.mdpi.com/article/10.3390/pharmaceutics13050634/s1, Supplementary Table S1: Overview of the experimental groups used in the biodistribution study of layer-encapsulated ^224^Ra surface-labeled CaCO_3_ microparticles with EDTMP added to control the size of microparticles, Supplementary Table S2: Overview of the experimental groups used in the study of the therapeutic efficacy of ^224^Ra-labeled CaCO_3_ microparticles, comparing the surface-labeled microparticles without EDTMP with the layer-encapsulated surface-labeled microparticles with added EDTMP, Supplementary Table S3: Summary of *p*-values in the biodistribution study obtained by performing a *t*-test on each pair of experimental groups, Supplementary Table S4: Summary of *p*-values in the two studies of therapeutic efficacy obtained from the Gehan–Breslow–Wilcoxon method, Supplementary Figure S1: Photomicrographs of CaCO_3_ microparticles, Supplementary Figure S2: Distribution of ^208^Pb and ^212^Pb on layer-encapsulated microparticles with 2.5% (*w*/*w*) EDTMP as a function of days after labeling with ^224^Ra, Supplementary Figure S3: Percentage adsorption of ^212^Pb^2+^ and ^212^Pb-EDTMP to nonradioactive mock-labeled CaCO_3_ MPs, surface-labeled MPs, and layer-encapsulated surface-labeled MPs.
