# Supplementary material for: Improved Formulation of 224Ra-Labeled Calcium Carbonate Microparticles by Surface Layer Encapsulation and Addition of EDTMP
Source: Pharmaceutics. 2021 Apr 29;13(5):634. doi: 10.3390/pharmaceutics13050634 (PMC8145685; doi:10.3390/pharmaceutics13050634)
Supplement: Supplementary file 1 [file pharmaceutics-13-00634-s001.zip › pharmaceutics-1193411-suppl.pdf]

pharmaceutics

Supplementary Materials: Improved Formulation of ^{224}Ra-Labeled Calcium Carbonate Microparticles by Surface Layer Encapsulation and Addition of EDTMP

Ruth Gong Li, Kim Lindland, Sandra Karen Tonstad, Tina Bjørnlund Bønsdorff, Asta Juzeniene, Sara Westrøm [1] and Roy Hartvig Larsen

Table S1. Overview of the experimental groups used in the biodistribution study of layer-encapsulated ^{224}Ra surface-labeled $CaCO_3$ microparticles with EDTMP added to control the size of microparticles. na: not applicable.

Group	EDTMP conc (% *w/w*)[(1)]	Median particle diameter (μm)	Radioactivity dose (kBq)	Mass dose (mg)	Age of mice (weeks)	Range of body weight (g)[(2)]	*n*
^{224}Ra-$CaCO_3$ MPs	1.2	7.6	6	1	7	22.8–26.5	3
^{224}Ra-$CaCO_3$ MPs	1.2	7.7	7	6	7	21.8–23.5	3
^{224}Ra-$CaCO_3$ MPs	2.5	7.0	9	5	7	20.0–23.3	3
^{224}Ra-$CaCO_3$ MPs	1.2	7.1	18	12	7	23.1–25.2	3
^{224}Ra-$CaCO_3$ MPs	1.5	8.8	13	12	10-13	24.1–30.0	3
^{224}RaCl_2	na	na	~30	na	7	20.8–23.9	3

[(1)] Relative to the mass of $CaCO_3$, [(2)] At the start of the study.

Table S2. Overview of the experimental groups used in the study of therapeutic efficacy of ^{224}Ra-labeled $CaCO_3$ microparticles, comparing surface-labeled microparticles without EDTMP with the layer-encapsulated surface-labeled microparticles with added EDTMP. na: not applicable.

Tumor cell line	Treatment	EDTMP conc (% *w/w*)[(1)]	Median particle diameter (μm)	Radioactivity dose (kBq)	Range of body weight (g)[(2)]	Mass dose (mg)	*n*
ES-2	Control (saline)	na	na	na	16.7–22.4	na	6
ES-2	^{224}Ra-$CaCO_3$ MPs Surface labeled	0	8.9	14	19.9–22.5	4	5
ES-2	^{224}Ra-$CaCO_3$ MPs Surface-labeled and layer-encapsulated	1.2	6.7	17	18.8–23.3	9	6
CT26.WT	Control (saline)	na	na	na	18.1–20.8	na	8
CT26.WT	^{224}Ra-$CaCO_3$ MPs Surface-labeled	0	22.7	26	18.8–21.6	5	8
CT26.WT	^{224}Ra-$CaCO_3$-MPs Surface-labeled and layer-encapsulated	1.2	6.6	22	18.5–21.2	14	8

[(1)] Relative to the mass of CaCO3, [(2)] At the start of the study.

Table S3. Summary of *p*-values in the biodistribution study obtained by performing a *t*-test on each pair of experimental groups. *p*-values have been adjusted by the Holm-Sidak method to include multiple comparisons.

**Radium-224**								
**Organ / Comparison**	**Blood**	**Kidneys**	**I.p. fat**	**Spleen**	**Liver**	**Lungs**	**Femur**	**Skull**
1 mg/$^{224}RaCl_2$	0.2921	0.7224	0.7224	0.2998	0.0601	0.7224	0.0035	0.0049
5–6 mg/$^{224}RaCl_2$	0.6041	0.6041	0.5131	0.6041	0.0444	0.2670	0.0001	0.0004
12 mg/$^{224}RaCl_2$	0.5850	0.4474	0.3195	0.6176	0.0052	0.1502	<0.00001	<0.0001
1 mg/12mg	0.1964	0.3458	0.3458	0.1037	0.0103	0.3458	0.0051	0.0075
1 mg/5–6 mg	0.6008	0.6008	0.6008	0.5373	0.0917	0.6008	0.5590	0.6008
5–6 mg/12 mg	0.3211	0.9099	0.9099	0.6816	0.4528	0.3380	0.1761	0.5277
**Lead-212**								
**Organ / Comparison**	**Blood**	**Kidneys**	**I.p. fat**	**Spleen**	**Liver**	**Lungs**	**Femur**	**Skull**
1 mg/$^{224}RaCl_2$	0.0009	0.4887	0.5439	0.3970	0.2739	0.5439	0.0010	0.0015
5–6 mg/$^{224}RaCl_2$	<0.0001	0.0014	0.1630	0.5678	0.8906	0.0088	0.0014	0.0014
12 mg/$^{224}RaCl_2$	<0.0001	0.0004	0.0684	0.7808	0.0127	0.0002	<0.0001	<0.0001
1 mg/12mg	0.0051	0.0107	0.3835	0.0837	0.0090	0.3186	0.0005	0.3186
1 mg/5–6 mg	0.2871	0.0334	0.5597	0.5597	0.1205	0.5597	0.6202	0.5597
5–6 mg/12 mg	0.4371	0.8112	0.4371	0.4371	0.4371	0.7462	0.2647	0.1573

Table S4. Summary of *p*-values in the two studies of therapeutic efficacy obtained from the Gehan-Breslow-Wilcoxon method. *p*-values have been adjusted by the Holm-Sidak method to include multiple comparisons.

**ES-2**			
**Study group**	**Control**	**$^{224}Ra-CaCO_3$-MPs Surface labeled**	$^{224}Ra-CaCO_3$-MPs Surface labeled and layer encapsulated
Control	-	0.0068	0.0042
$^{224}Ra-CaCO_3$ MPs Surface-labeled	0.0068	-	0.4477
$^{224}Ra-CaCO_3$ MPs Surface-labeled and layer- encapsulated	0.0042	0.4477	-
**CT26.WT**			
**Study group**	Control	$^{224}Ra-CaCO_3$ MPs Surface-labeled	$^{224}Ra-CaCO_3$ MPs Surface-labeled and layer-encapsulated
Control	-	0.044	0.044
$^{224}Ra-CaCO_3$ MPs Surface-labeled	0.044	-	0.6331
$^{224}Ra-CaCO_3$ MPs Surface-labeled and layer- encapsulated	0.044	0.6331	-

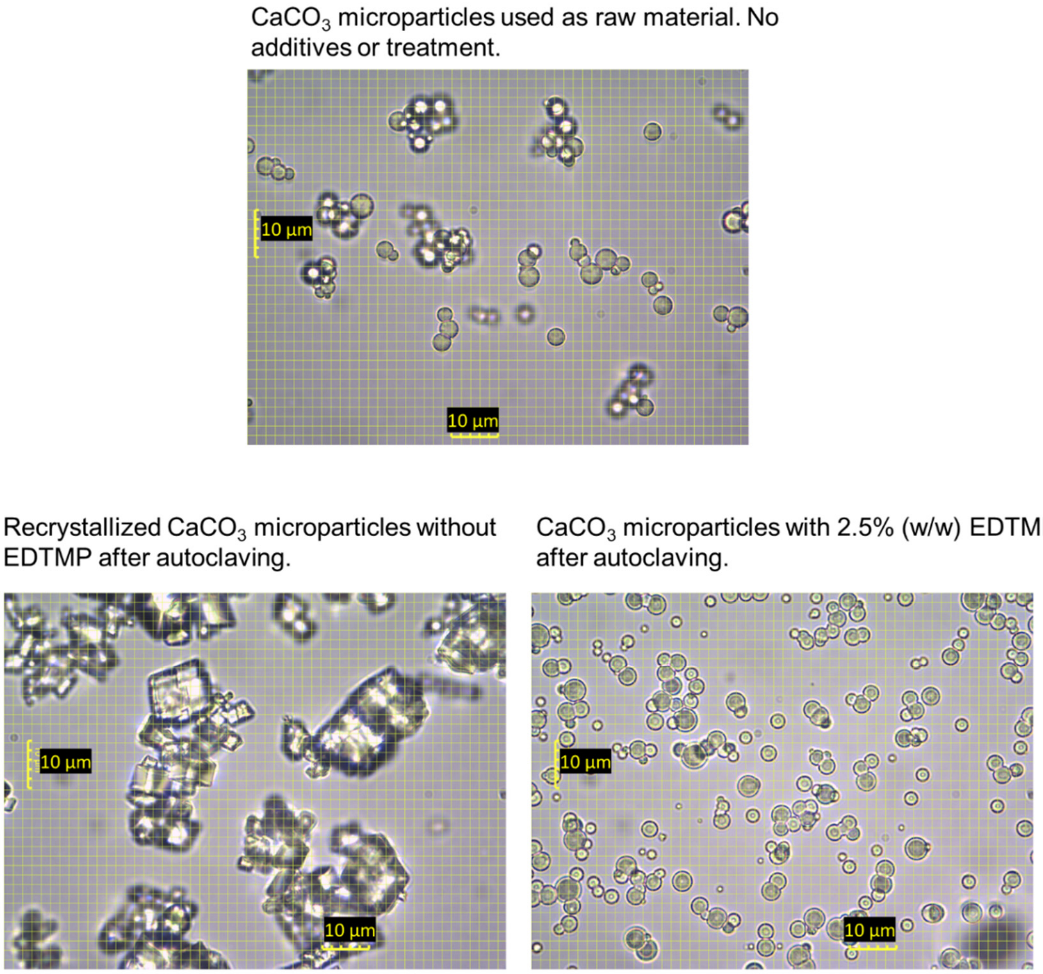

Figure S1. Photomicrographs of $CaCO_3$ microparticles. The mesh size indicates 2 µm.

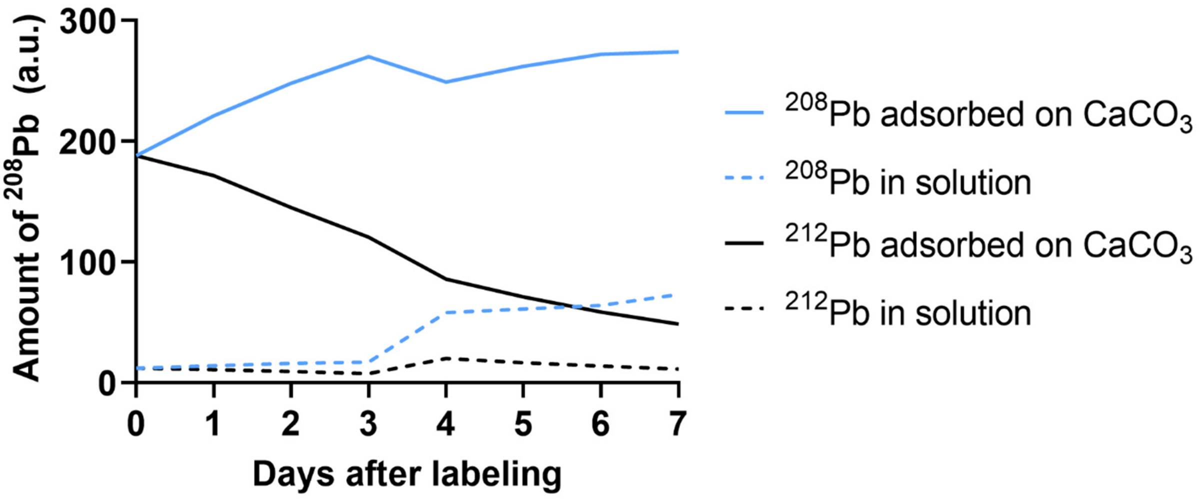

Figure S2. Distribution of ^{208}Pb and ^{212}Pb on layer-encapsulated microparticles with 2.5% *(w/w)* EDTMP as a function of days after surface labeling with ^{224}Ra. It is assumed that ^{224}Ra is in equilibrium with its daughters, with a total amount of 200 atoms (or a.u.) for each of ^{224}Ra, ^{212}Pb, and ^{208}Pb on day zero. An equal proportion of ^{208}Pb and ^{212}Pb adsorbed on MPs (RCP) versus in the solution, is assumed, with 94% RCP from day 0–3, 81% RCP from day 4–6, and 79% RCP on day 7. One ^{208}Pb atom is produced per ^{224}Ra decay, while the total number of ^{212}Pb atoms is found by using the Bateman equation, taking both ^{224}Ra and ^{212}Pb decay into account.

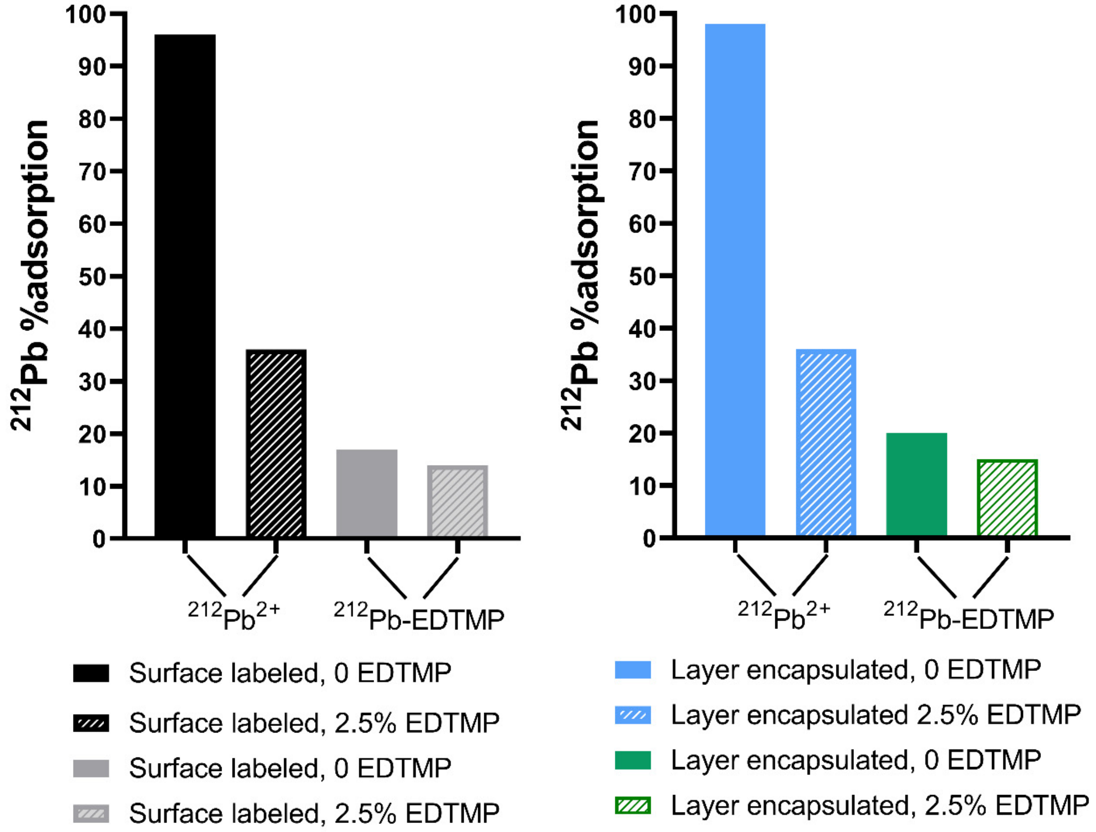

Figure S3. Percentage adsorption of $^{212}Pb^{2+}$ and ^{212}Pb-EDTMP to nonradioactive mock-labeled $CaCO_3$ MPs, surface- labeled MPs and layer-encapsulated surface-labeled MPs. EDTMP concentrations indicate the relative concentration in the MP suspension with respect to grams per gram of $CaCO_3$.
